# Using Principles of Digital Development for a Smartphone App to Support Data Collection in Patients With Acute Myocardial Infarction and Physical Activity Intolerance: Case Study

**DOI:** 10.2196/33868

**Published:** 2024-03-18

**Authors:** Diana Isabel Cáceres Rivera, Luz Mileyde Jaimes Rojas, Lyda Z Rojas, Diana Canon Gomez, David Andrés Castro Ruiz, Luis Alberto López Romero

**Affiliations:** 1 Facultad de Enfermería Universidad Cooperativa de Colombia Bucaramanga Colombia; 2 Centro de Investigaciones Fundación Cardiovascular de Colombia Floridablanca Colombia; 3 Departamento de Pediatría, de Obstetricia y Ginecología y de Medicina Preventiva y Salud Pública Universidad Autónoma de Barcelona Barcelona Spain

**Keywords:** app, applications of medical informatics, coronary disease, data collection, development, health care reform, health data, medical informatics, medical informatics apps, mobile app, mobile applications, nursing diagnosis, nursing research, research data, software, validation

## Abstract

**Background:**

Advances in health have highlighted the need to implement technologies as a fundamental part of the diagnosis, treatment, and recovery of patients at risk of or with health alterations. For this purpose, digital platforms have demonstrated their applicability in the identification of care needs. Nursing is a fundamental component in the care of patients with cardiovascular disorders and plays a crucial role in diagnosing human responses to these health conditions. Consequently, the validation of nursing diagnoses through ongoing research processes has become a necessity that can significantly impact both patients and health care professionals.

**Objective:**

We aimed to describe the process of developing a mobile app to validate the nursing diagnosis “intolerance to physical activity” in patients with acute myocardial infarction.

**Methods:**

We describe the development and pilot-testing of a mobile system to support data collection for validating the nursing diagnosis of activity intolerance. This was a descriptive study conducted with 11 adults (aged ≥18 years) who attended a health institution for highly complex needs with a suspected diagnosis of coronary syndrome between August and September 2019 in Floridablanca, Colombia. An app for the clinical validation of activity intolerance (North American Nursing Diagnosis Association [NANDA] code 00092) in patients with acute coronary syndrome was developed in two steps: (1) operationalization of the nursing diagnosis and (2) the app development process, which included an evaluation of the initial requirements, development and digitization of the forms, and a pilot test. The agreement level between the 2 evaluating nurses was evaluated with the κ index.

**Results:**

We developed a form that included sociodemographic data, hospital admission data, medical history, current pharmacological treatment, and thrombolysis in myocardial infarction risk score (TIMI-RS) and GRACE (Global Registry of Acute Coronary Events) scores. To identify the defining characteristics, we included official guidelines, physiological measurements, and scales such as the Piper fatigue scale and Borg scale. Participants in the pilot test (n=11) had an average age of 63.2 (SD 4.0) years and were 82% (9/11) men; 18% (2/11) had incomplete primary schooling. The agreement between the evaluators was approximately 80% for most of the defining characteristics. The most prevalent characteristics were exercise discomfort (10/11, 91%), weakness (7/11, 64%), dyspnea (3/11, 27%), abnormal heart rate in response to exercise (2/10, 20%), electrocardiogram abnormalities (1/10, 9%), and abnormal blood pressure in response to activity (1/10, 10%).

**Conclusions:**

We developed a mobile app for validating the diagnosis of “activity intolerance.” Its use will guarantee not only optimal data collection, minimizing errors to perform validation, but will also allow the identification of individual care needs.

## Introduction

In recent decades, the ability to produce, collect, and communicate data around the world has increased exponentially with access to technologies such as smartphones. These technologies have improved data storage as well as its handling and analysis [1]. In the field of health, electronic record systems facilitate data collection that can be used for various purposes, allowing data retrieval that promotes the improvement of research processes such as identification and recruitment of patients for clinical projects [2,3].

In addition to obtaining individual data from each patient, the collection of large amounts of data can be useful to obtain information that more effectively supports the exploration of diseases, treatment, and rehabilitation. This creates the need to develop research platforms that optimize the capacity to conduct informative and innovative research and enable scientific approaches where objective data can be obtained with a minimum of errors and expended resources [4].

As part of the health staff providing care to cardiovascular patients, nurses can be the first to identify individual needs. To aid this, tools are available such as the NANDA (North American Nursing Diagnosis Association) taxonomy, which identifies the response of a person, family, or community to real health problems and potential vital processes. However, these diagnoses and their respective defining characteristics must be validated according to the context where they will be assessed, which constitutes a challenge in research into the use, implementation, and dissemination of technologies of information [5-7]. For this purpose, the use of digital platforms has demonstrated its applicability from the early stages of research, such as the assessment of care needs [8,9].

Mobile apps in health, education, and work in Colombia are promoting efficient new practices to streamline processes and improve access to information at the national level, with the intention of contributing to the modernization and globalization of different socioeconomic sectors. These technologies are important to innovate in the health sector because they can benefit both patients and health staff. However, the uptake of this type of technological tool is still slow and limited [10]. Thus, this paper describes the process of developing a mobile app for collecting health research data. Specifically, it is intended that this app will be a tool that allows speeding up the validation of a nursing diagnosis in an objective and practical way.

## Methods

### Overview

This was a descriptive study conducted with 11 adults (aged ≥18 years) with a suspected diagnosis of coronary syndrome who attended a health institution for highly complex needs between August and September 2019 in Floridablanca, Colombia. An app for clinical validation of the “activity intolerance” diagnosis (NANDA code 00092) in patients with acute coronary syndrome was developed in three steps, outlined in the following sections.

### Step 1: Operationalization of the Nursing Diagnosis

The first step consisted in the operationalization of the defining characteristics of the nursing diagnosis [11] of activity intolerance (NANDA code 00092), defined by NANDA-I [6] as “the lack of sufficient physiological or psychological energy to tolerate or complete the required or desired daily activities.” This diagnosis is categorized as “Domain 4: Activity / Rest, Class 4: Cardiovascular / pulmonary responses Need: Move and Pattern Activity-exercise.” It is also related to an imbalance between oxygen supply and demand, a sedentary lifestyle, immobility, and bed rest; it has defined characteristics [12]. Through an extensive search of the literature, we selected scales or instruments to standardize the measurement of each defining characteristic of this nursing diagnosis [11]. An interdisciplinary group that included 2 nurses, an epidemiologist, and a cardiologist verified the face validity of the operationalization.

### Step 2: App Development Process

#### Initial Requirements Evaluation

Health professionals, along with a systems engineer, carried out the structural design of the data collection forms or case report forms. The digitization process was carried out using CommCare [7], which is an open source, cloud-based platform that helps researchers develop data capture tools using mobile devices. An open source tool was also used to create an Android-based mobile app for a low-income setting. Mobile apps can be used as a tool to track beneficiaries through a service lifecycle and can also streamline data collection [13]. Our app used the HTTPS protocol, which made it cryptographically secure. Access to data was password protected. The CommCare [7] platform was selected because it has been widely used for health projects all over the world and because of its ease of use and compatibility with older versions of Android. CommCare is a platform that works on Android mobile phones from version 2.3, but the platform recommends reviewing the documentation for these older versions because they may have limitations in terms of functionality and compatibility with the latest features developed by CommCare, so it is recommended to have at least Android version 4.0.3 or later, a storage space of at least 100MB, a minimum of 1GB of RAM, and a processor with at least 2 cores for a better user experience.

**Figure 1:**
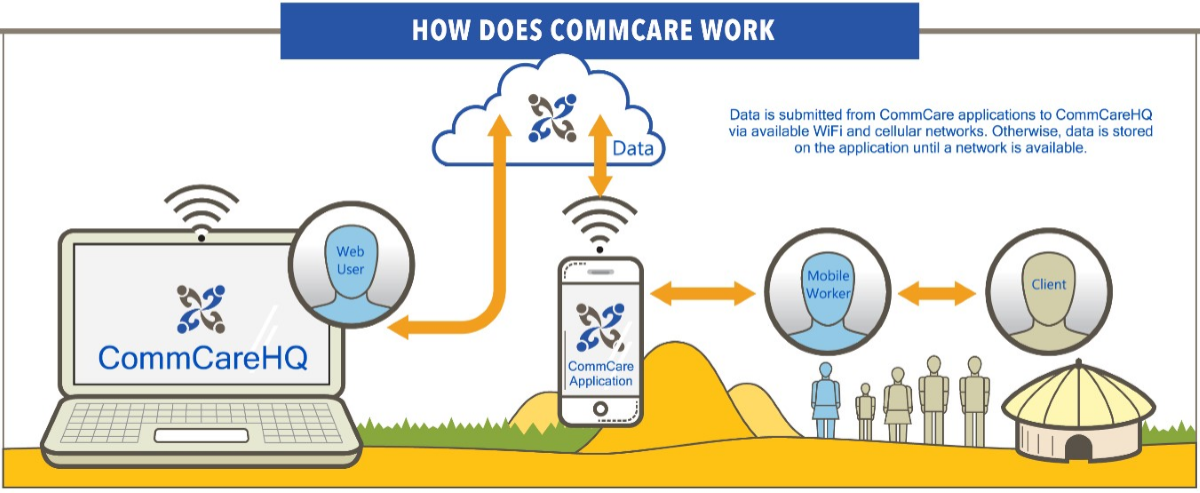
Commcare platform design.

Finally, we did not use any programming language because we used a platform that prevents us from reaching that level. We worked directly with CommCare, which allowed us to create data collection applications without touching or programming source code (Figure 1).

CommCare requires the use of a password to access the app and the data stored on the platform. This helps to ensure that only authorized users can access information. The platform uses the secure HTTPS communications protocol, uses role-based access, and is in compliance with data security regulations and standards such as the European Union’s General Data Protection Regulation (GDPR). This ensures that the platform follows good practices in terms of privacy and personal data protection.

#### Development and Digitization of the Forms

The principles of the Scrum methodology for agile software development were applied. This is a regularly applied process that includes a set of best practices to work collaboratively in teams and obtain the best possible outcome of projects. It is characterized by a strategy of incremental development, boosting the quality of the result by getting to know people in self-organized teams and matching the different phases of development, rather than doing one after the other in a sequential or cascading cycle [14]. Through this methodology, an app was developed to gather data. This phase included the following six steps: (1) specifying the forms to be digitized, which contained the questions or variables to be obtained in the field; (2) dividing the various sections of the form into smaller subforms, depending on the size of the questionnaire or the time of application; (3) defining the variables as the simple question-and-answer type or as more complex ones containing calculations, depending on others, or having a different logical flow; (4) building the form on the CommCare platform; (5) generating app versions (eg, test versions); and (6) testing the app with health professionals who simulated data from possible patients and followed the flow of questions within the app to check if the different flows worked correctly; if errors or possible improvements were found during the process, the entire procedure was repeated from step 4.

### Ethical Considerations

The Ethics Committee of Universidad Cooperativa de Colombia thoroughly reviewed and approved the research (report 003; April 16, 2018), as did the Fundación Cardiovascular de Colombia ethics committee (report 450; May 22, 2018). The study was carried out in strict adherence to the established protocol, regulatory requirements, Good Clinical Practice, the Declaration of Helsinki, and the clinical investigation guidelines of Universidad Cooperativa de Colombia. All participants provided their informed consent by signing a form. Participation in this study was entirely voluntary, and no financial compensation or reimbursements were offered to the participants.

The information obtained has been securely stored in the archives of the Universidad Cooperativa de Colombia to safeguard the privacy of individuals. Each patient was assigned a code to ensure that their names or identification did not appear in the database. Access to the collected data was restricted to the researchers, and the data will be used exclusively for the study’s intended purposes. Personal information is being protected in compliance with Colombian Law 1581 of 2012, which pertains to the right of “habeas data.”

## Results

After repeatedly performing the entire process and correctly digitizing all the forms proposed in advance, the last version of the app (the production version) was generated. The result of the development process was an app that allowed obtaining information using the forms shown in Table 1.

Table 2 shows the scales and instruments used for the operationalization of the defining characteristics of nursing.

The resulting app allowed the simultaneous collection, data entry, and follow-up of patients in different stages of investigation. Two previously trained nurses conducted a pilot test with the first 11 patients included in the research. Taking into account the inclusion and exclusion criteria, a cardiologist selected potential patients. Subsequently, the patient received an explanation of the study; if they agreed to participate, they provided informed written consent. The information was filled out on tablet-type mobile device. Once the data were collected, a process of sending or synchronizing the data with the database in the cloud was carried out, for which it was necessary to have an internet connection (Wi-Fi network).

**Table 1:** General information included in the app.

Information collected	Forms used
Sociodemographic data	Personal data of the patient
Registered patient forms	Information about the patient
Hospital admission data	Referral or admission information
Background	Medical, surgical, family, and toxicology history
Current drug treatment	Angiotensin-converting enzyme inhibitors, statins, β-blockers, angiotensin II receptor blockers, aldosterone antagonists, acetylsalicylic acid, diuretics, thiazide diuretics, digitalis, antiplatelet agents, anticoagulants, vasodilators, antiarrhythmics, analgesics, inotropics
Intensity of angina	Angina intensity level
Diagnostic means	Electrocardiogram, electrographic changes, electrocardiogram findings, arteriography findings, cardiac enzymes, and other paraclinical tools
TIMI-RS^a^	TIMI-RS (S-elevation and non-ST elevation; acute myocardial infarction/unstable angina)
GRACE^b^ score	GRACE score
Defining characteristics of the diagnosis	Electrocardiogram changes, generalized weakness, exertional discomfort, dyspnea on exertion, fatigue, abnormal heart rate in response to activity, and abnormal blood pressure in response to activity
Follow-up	Presence of fatigue, pain, dyspnea on exertion, and physical activity

^a^TIMI-RS: thrombolysis in myocardial infarction risk score.

^b^GRACE: Global Registry of Acute Coronary Events.

**Table 2:** Scales and validated instruments included in the app for diagnosing intolerance to activity.

Defining characteristics	Scale or instrument used
Electrocardiogram changes (arrhythmias, conduction abnormality, ischemia)	Suggested changes by Clinical Practice Guideline of the Ministry of Social Protection of Colombia [15]
General weakness	Handgrip measurement (muscle strength in kg) and bioimpedance measurement
Exertional discomfort	Brief Disease Perception Questionnaire [16]
Exertional dyspnea	Grade of dyspnea according to the New York Heart Association [17]
Fatigue	Piper Fatigue Scale [18]; Ruffier test (aerobic capacity) [19]; Borg scale (perceived physical exertion) [20]
Abnormal heart rate in response to activity	The suggested values in the Clinical Practice Guide of the Ministry of Social Protection of Colombia [14]
Abnormal blood pressure in response to activity	2018 European Society of Cardiology/European Society of Hypertension Clinical Practice Guidelines for the Management of Arterial Hypertension [21]

**Figure 2:**
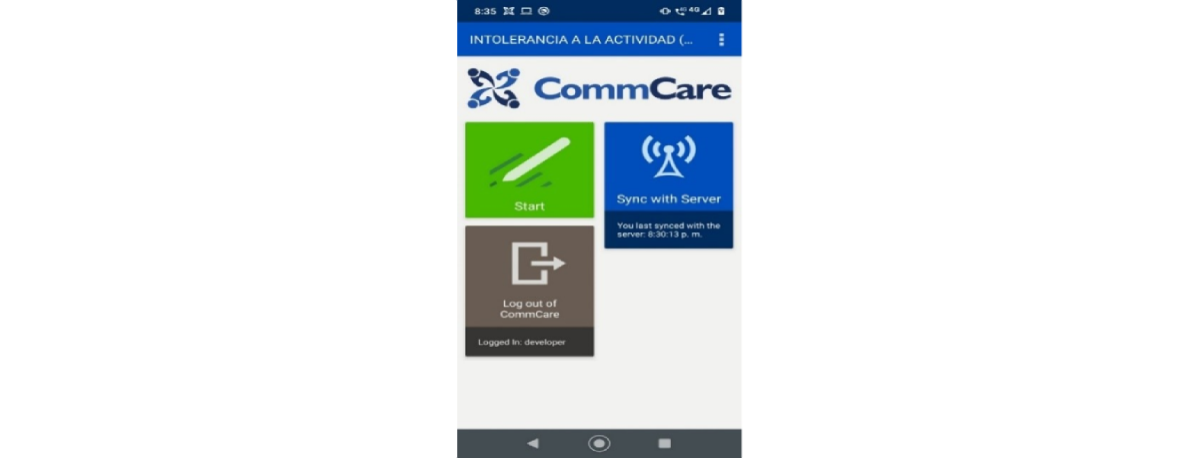
Screenshot of app start screen.

**Figure 3:**
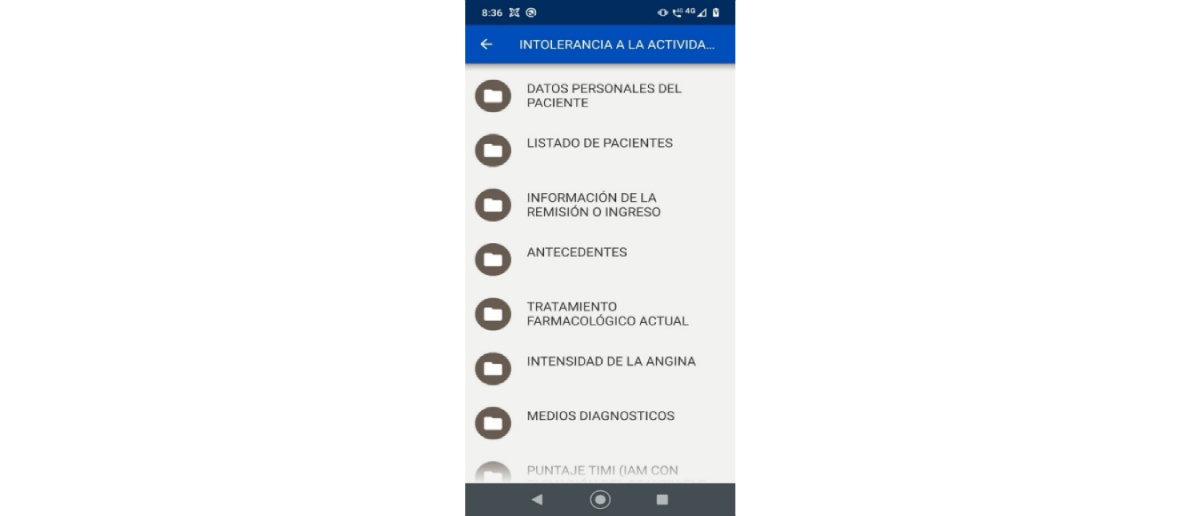
Screenshot showing the list of forms that can be used with patients in the field.

**Figure 4:**
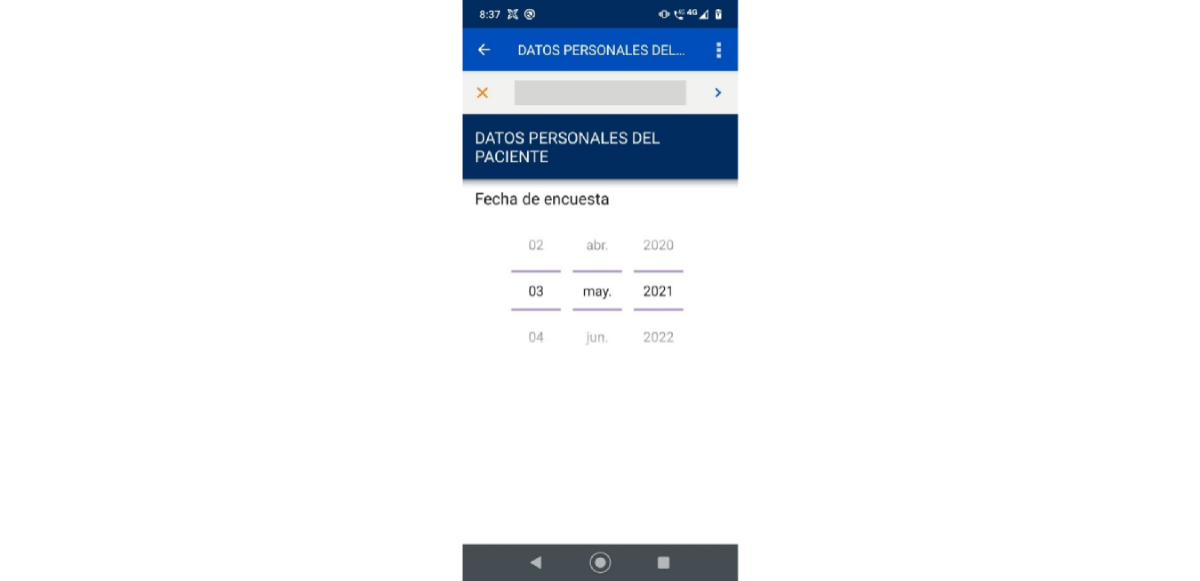
Screenshot showing question on the personal data of the patient.

Later, the systems engineer reviewed the database obtained through the web platform CommCare, which allows downloading information as a flat file or in spreadsheet format. In this way, the research team verified the correct operation of the app and its use in the field, obtaining positive results that allowed the continuity of the investigation with more patients. Figures 2-4 show 3 screenshots of the app.

The pilot test yielded descriptive data (n=11). The participants had an average age of 63.2 (SD 4.0) years, 82% (9/11) were men, and 18% (2/11) had incomplete primary schooling. We found that 64% (7/11) had a history of hypertension and 73% (8/11) had ever smoked. The defining characteristics present in this group of patients were exercise discomfort in 91% (10/11), electrocardiogram abnormalities in 9% (1/10), abnormal heart rate in response to exercise in 20% (2/10), dyspnea in 27% (3/11), weakness in 64% (7/11) and abnormal blood pressure in response to activity in 10% (9/10) (Table 3). The κ agreement index ranged from 73% to 100%.

**Table 3:** Presence of defining characteristics of the nursing diagnosis “activity intolerance” in patients (n=11) with acute coronary syndrome according to 2 independent evaluators.

Defining characteristics	Participants evaluated by nurse 1, n (%)			Participants evaluated by nurse 2, n (%)		Agreement between the evaluators, %
	Yes	No	Yes		No	
Electrocardiogram changes (arrhythmias, conduction abnormality, ischemia)	1 (9)	10 (91)	0 (0)		11 (100)	91
General weakness	7 (64)	4 (36)	8 (73)		3 (27)	73
Exertional discomfort	10 (91)	1 (9)	9 (82)		2 (18)	73
Exertional dyspnea	3 (27)	8 (73)	3 (27)		8 (73)	100
Fatigue	0 (0)	11 (100)	0 (0)		11 (100)	0
Abnormal heart rate in response to activity	2 (20)	8 (80)	2 (20)		8 (80)	100
Abnormal systolic blood pressure in response to activity	1 (10)	9 (90)	0 (0)		10 (100)	90
Abnormal diastolic blood pressure in response to activity	0.0 (0)	10 (100)	1 (10)		9 (90)	90

## Discussion

### Principal Findings

We describe the development process of a mobile app for collecting health research data in an easy, agile, and practical way. This strategy may be used for the complete collection of samples in the process of clinical validation of the nursing diagnosis “activity intolerance.” In addition, a good rate of agreement was found among the evaluators thanks to the standardization used in the app.

In recent years there has been an increase in the use of computer technologies to replace paper records by means of mobile apps, web forms, and specialized software; likewise, it has become evident that these are key tools to improve quality in health care [22]. However, it is still a challenge to continue implementing new strategies, achieve their efficient use by health professionals, and make their implementation easier and more accessible.

This process enabled us to validate the app’s use for identifying prevalent nursing diagnoses, such as activity intolerance, in patients with acute myocardial infarction. Among the 9 defining characteristics we evaluated, there was an agreement of over 80% among the evaluators for 5 of them. This, in turn, helped us identify the most prevalent characteristics, namely dyspnea on exertion and heart rate alteration in response to activity. It is also noteworthy that none of the evaluators identified fatigue in any of the users.

### Mobile Apps

We evaluated this strategy for identifying nursing diagnoses that require an objective definition of their characteristics and clinical judgment [23]. The precise operationalization of the defining characteristics through a predefined registry structure, as seen in this mobile app, enhances the precision of nursing diagnoses [1]. In this sense, it enables the evaluation of these characteristics, which can improve documentation for nursing staff, thereby aiding in the inference and evaluation of diagnoses [2]. Therefore, this app aims not only to enhance the quality and safety of care processes but also to promote the adoption of standardized nursing language, addressing the limitations in its use.

Another possible use of this app is in education, where it would potentially help to strengthen the precision of documentation in nursing diagnoses [3]. This strategy is adapted to current conditions, in which the use of virtual methods and mobile technologies has been shown to be a new basic input for the teaching process, making it necessary for professionals and trainers to make an adequate use of this type of strategy.

A relationship where nurse and patient can contribute to improving administrative processes that benefit others has been described in settings such as outpatient care [5]. This is expected to contribute to research scenarios that promote improved caregiving. Apps can assist in the assessment and generation of nursing diagnoses in hospital practice [24], and they have been used in research studies such as clinical trials for the self-management of angina [25].

### Limitations

This work was limited to a specific nursing diagnosis. Future work should include other prevalent diagnoses in patients with cardiac disease. An evaluation of usability among end users could help improve our strategy, and more data is also needed to better specify the large-scale feasibility and cost of this strategy with other nursing diagnoses.

Other aspects to improve in the design of future research are to include scales and instruments used in health care to measure different variables. These sources of information should be updated according to the context, clinical conditions, and even environmental conditions. An additional challenge is the integration of these types of apps to existing health systems. A recent review with the objective to provide an overview of studies that have collected patient data using an app-based approach indicated that using mobile technologies could help to overcome challenges linked with data collection in epidemiological research. However, further feasibility studies need to be conducted to test the applicability and acceptance of these mobile apps for epidemiological research in various subpopulations [26].

### Conclusions

We developed a mobile app for use in the validation process of the nursing diagnosis activity intolerance. This app enabled the evaluation of defining characteristics, which can enhance documentation for nursing staff, facilitate more effective inference and evaluation of diagnoses, and reduce errors in information recording. One significant potential of this app lies in its impact on education, as it aids in improving the precision of nursing diagnosis documentation and, as a result, enhances the quality of care planning.

## Abbreviations

GDPR: General Data Protection Regulation
NANDA: North American Nursing Diagnosis Association

## Data Availability

The data sets generated during and/or analyzed during this study are available in the Mendeley repository [27].

## References

[1] Carrillo G, Mesa Y (2007). La investigación en validación de diagnósticos de enfermería. Rev Cubana Enferm.

[2] van Dam Joris, Omondi Onyango Kevin, Midamba B, Groosman N, Hooper N, Spector J, Pillai GC, Ogutu B (2017). Open-source mobile digital platform for clinical trial data collection in low-resource settings. BMJ Innov.

[3] Style S, Beard BJ, Harris-Fry H, Sengupta A, Jha S, Shrestha BP, Rai A, Paudel V, Thondoo M, Pulkki-Brannstrom A, Skordis-Worrall J, Manandhar DS, Costello A, Saville NM (2017). Experiences in running a complex electronic data capture system using mobile phones in a large-scale population trial in southern Nepal. Glob Health Action.

[4] Martinez AD, Salazar C (2018). Impacto de las aplicaciones móviles en Colombia a nivel de la salud, educación y trabajo. Fund Univ Católica Lumen Gentium.

[5] Martín FA, Marco CG, Antonio SOJ (2020). Evaluation and acreditation of mobile health applications. Rev Esp Salud Publica.

[6] NANDA Internacional (2021). Diagnósticos Enfermeros. Definiciones y Clasificación. 2021-2023.

[7] CommCare.

[8] Bissi W (2007). Metodologia De Desenvolvimento Ágil. Campo Digit.

[9] Kerwin TC, Leighton H, Buch K, Avezbadalov A, Kianfar H (2016). The effect of adoption of an electronic health record on duplicate testing. Cardiol Res Pract.

[10] Rojas Sánchez Lyda Zoraya, Hernández Vargas Juliana Alexandra, Trujillo Cáceres Silvia Juliana, Roa Díaz Zayne Milena, Jurado Arenales AM, Toloza Pérez Yesith Guillermo (2017). Usefulness of the diagnosis "decreased cardiac output (00029)" in patients with chronic heart failure. Int J Nurs Knowl.

[11] Orozco-Vargas LC, Santander UID (2010). Validez y validación o de cómo construir la validez de un constructo. Medición en salud: Diagnóstico y evaluación de resultados. Un manual crítico más allá de lo básico.

[12] Paans W, Sermeus W, Nieweg RM, Krijnen WP, van der Schans CP (2012). Do knowledge, knowledge sources and reasoning skills affect the accuracy of nursing diagnoses? a randomised study. BMC Nurs.

[13] Facione N, Facione PA (2006). The Health Sciences Reasoning Test.

[14] Baraki Z, Girmay F, Kidanu K, Gerensea H, Gezehgne D, Teklay H (2017). A cross sectional study on nursing process implementation and associated factors among nurses working in selected hospitals of Central and Northwest zones, Tigray Region, Ethiopia. BMC Nurs.

[15] (2013). Guía de práctica clínica para El Síndrome Coronario Agudo. Sistema General de Seguridad Social en Salud, Colombia.

[16] Bazán Riverón GE, Osorio Guzmán M, Miranda AL, Alcántara Vázquez O, Uribe Ortiz G (2013). Validación del cuestionario breve sobre percepción de la enfermedad (BIPQ) en hipertensos. Revista De Psicología (Trujillo).

[17] Clasificación de insuficiencia cardíaca de la New York Heart Association (NYHA). Manual MSD.

[18] Lamino DDA, Andruciolli de Mattos C, Braga PE, Corrêa de Faria Mota DD (2014). Fadiga clinicamente relevante em mulheres com câncer de mama: prevalência e fatores associados. Investg Enferm Imagen Desarollo.

[19] Alahmari KA, Rengaramanujam K, Reddy RS, Samuel PS, Kakaraparthi VN, Ahmad I, Tedla JS (2020). Cardiorespiratory fitness as a correlate of cardiovascular, anthropometric, and physical risk factors: using the Ruffier test as a template. Can Respir J.

[20] Williams Nerys (2017). The Borg Rating of Perceived Exertion (RPE) scale. Occup Med.

[21] (2013). Guía de práctica clínica: Hipertensión arterial primaria (HTA). Vol. 18, Guía No. 18. Ministerio de Salud y Protección Social-Colciencias.

[22] Lunney M (2008). Critical need to address accuracy of nurses’ diagnoses. Online J Issues Nurs.

[23] De Groot K, Sneep EB, Paans W, Francke AL (2021). Patient participation in electronic nursing documentation: an interview study among community nurses. BMC Nurs.

[24] Melo EBMD, Primo CC, Romero WG, Sant'Anna Hugo Cristo, Sequeira CADC, Lima EDFA, Fioresi M (2020). Construction and validation of a mobile application for development of nursing history and diagnosis. Rev Bras Enferm.

[25] Wang W, Chan S, He H (2014). Developing and testing a mobile application programme to support self-management in patients with stable angina: a feasibility study protocol. Stud Health Technol Inform.

[26] Fischer F, Kleen S (2021). Possibilities, problems, and perspectives of data collection by mobile apps in longitudinal epidemiological studies: scoping review. J Med Internet Res.

[27] Cáceres Diana Using the principles for digital development. Mendeley Data.

